# Perch and Its Parasites as Heavy Metal Biomonitors in a Freshwater Environment: The Case Study of the Ružín Water Reservoir, Slovakia

**DOI:** 10.3390/s120303068

**Published:** 2012-03-06

**Authors:** Tímea Brázová, Jordi Torres, Catarina Eira, Vladimíra Hanzelová, Dana Miklisová, Peter Šalamún

**Affiliations:** 1 Institute of Parasitology, Slovak Academy of Sciences, Hlinkova 3, 04001 Košice, Slovakia; E-Mails: hanzel@saske.sk (V.H.); miklis@saske.sk (D.M.); salamun@saske.sk (P.S.); 2 Laboratory of Parasitology, Faculty of Pharmacy, University of Barcelona, Av. Joan XXIII, s/n, 08028 Barcelona, Spain; E-Mail: jtorres@ub.edu; 3 CESAM & Departamento de Biologia, Universidade de Aveiro 3810-193 Aveiro, Portugal; E-Mail: catarina.eira@ua.pt

**Keywords:** heavy metals, fish, parasites, bioaccumulation factors, experiment

## Abstract

Heavy metal concentrations were determined in 43 perches (*Perca fluviatilis*) and in two of its most common parasites, the acanthocephalan *Acanthocephalus lucii* and the cestode *Proteocephalus percae*, collected in the period 2009–2010 from Ružín, a seriously polluted water reservoir in Slovakia. Samples of muscle, liver, kidney, brain, male and female reproductive organs and adipose tissue of fish and both parasites were analyzed for As, Cd, Cr, Cu, Hg, Mn, Ni, Pb and Zn, by ICP-MS. Mean concentrations of individual heavy metals in all fish samples decreased in the order zinc > copper > manganese > mercury > arsenic > chromium > cadmium > nickel > lead. Zinc was found to be the dominant element and its antagonistic interaction with copper was confirmed. The kidney was a key target organ receiving the highest mean concentrations of all analyzed metals, but some metals showed specific affinity for particular tissues. In terms of human health, concentration of Hg in fish muscle, which exceeded more than two-times its maximum level admitted in foodstuffs in European countries, is of great importance and should be taken into account. Bioaccumulation factors (C_[parasite]_/C_[fish tissue]_) calculated for all elements indicated much higher detection skills of *A. lucii* and *P. percae* parasites than fish organs and hence, present results allow proposing both parasite models as useful tools to monitor aquatic environmental quality. Acanthocephalans, however, seem to be superior for heavy metal monitoring, also demonstrated under experimental conditions. Present results also indicate the decreasing heavy metal burden of the reservoir and its gradual recovery in the course of time.

## 1. Introduction

Nowadays, there is no doubt that the environment receives large quantities of pollutants as a consequence of human activities and that those substances can have detrimental effects on humans as well as on the health of all living organisms. One of the most serious problems is pollution by heavy metals [1]. They occur naturally in the Earth’s crust and therefore are found in soils and rocks with subsequent concentrations in sediments, water and organisms. In addition to geological weathering, also anthropogenic releases from ore mines, metallurgical industry and other intensive mining activities can give rise to higher contamination of heavy metals in comparison to normal background values [2].

According to the Dangerous Substances Directive of the European Union [3] dangerous chemicals are defined as toxic, persistent and/or bioaccumulative substances. Unlike the majority of organic pollutants, which eventually degrade to carbon dioxide and water, heavy metals, as elements, cannot be broken down, and therefore, they persist in the environment and tend to accumulate especially in lake, estuarine or marine sediments [4]. Aquatic organisms have the ability to accumulate heavy metals from their surroundings across the gills due to respiration, and by ingestion of contaminated food [5,6]. The uptake and circulation of heavy metals via the food chain is very important because metals induce biochemical reactions in organisms with typical responses such as inhibition of growth, suppression of oxygen consumption and impairment of reproduction and tissue repair [7].

Biomonitoring is a vital and rapidly growing field that uses several biological groups such as phytoplankton, macrophytes, invertebrates and fish, as bioindicators [8]. This ecological approach is also increasingly important in assessing heavy metal loads in aquatic environments [9–12]. Nonetheless, a better understanding of harmful metal exposures and their biological effects on relevant receptors is still required [13,14]. Fish parasites are listed among species that have been identified as highly sensitive either because of their physiological response to aquatic contaminants or because of their ability to accumulate particular toxic agents [11,12,15]. In fact, certain intestinal parasites, particularly thorny-headed worms parasitizing fish, have been shown as organisms that accumulate heavy metals at concentrations that are many times higher than those recorded in the host tissues [11,16]. While acanthocephalans are most frequently used in aquatic ecotoxicological studies [11,17,18], representatives of other groups of helminths (e.g., cestodes, nematodes) rarely appear in similar studies [19,20].

This work was performed to assess the current state of pollution in the Ružín water reservoir, which has suffered severe contamination by heavy metals by comparing their concentrations in several perch tissues as well as in their parasites. We determined the concentrations of nine heavy metals explaining their tissue-specific distribution in the perch (*Perca fluviatilis*) and in their most common parasites (the acanthocephalan *Acanthocephalus lucii* and the cestode *Proteocephalus percae*). An experiment was conducted to compare the metal uptake by these two parasite species. Cadmium was chosen for experimental design as the model metal, because of its highly effective accumulation into the body of parasites. Despite the lowest Cd concentration of all elements assessed in bottom sediments of the water reservoir (Table 1), in both parasites Cd was found in relatively high quantities. as also documented individual bioconcentration factors (BF = C_[parasite]_/C_[host tissue]_) calculated for this element. We also assessed the possible changes of environmental conditions since mining and metallurgical activities were ceased in the area ten years ago.

## 2. Materials and Methods

### 2.1. Study Site

**Figure 1.**
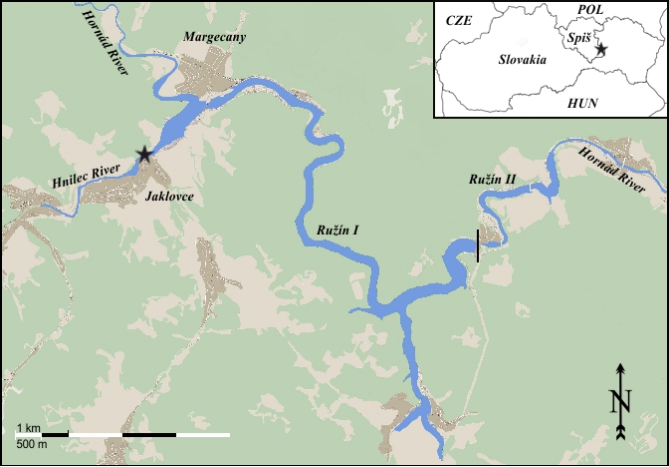
The Ružín water reservoir with indication of the sampling site (★) located near Jaklovce village and the mouth of the Hnilec River into the reservoir.

The Ružín water reservoir, with an area of 3.9 km^2^ and a water volume of 59 million m^3^ is located in eastern Slovakia (48°40′N, 20°53′E) (Figure 1). It was created by damming the Hornád River in 1967 (Ružín I) and 1972 (Ružín II). Ružín receives waters from the Hornád and Hnilec Rivers, which drain the Spiš-Gemer Rudohorie Mts. [21].

This territory was historically known for intensive mining and ore processing activities. Bottom sediments of the reservoir have high concentrations of heavy metals [21] originating from the geology and rock environment of the area and in the processing of ore and mining materials from the sites located near Krompachy, Rudňany and the surroundings of Spišská Nová Ves and Smolník towns (Table 1). A proportion of contamination is due to atmospheric deposition. The increased moisture above the reservoir’s water surface induces the coagulation of particles (ions, molecules, molecular aggregates, inorganic particles themselves) that fall on water and are stored in bottom sediments. The highest quantities of heavy metals were found near the mouth of the rivers into the reservoir [22].

The rich ichthyofauna of the reservoir (both in terms of species diversity and population density) is made up mainly by common carp *Cyprinus carpio* L., pike-perch *Sander lucioperca* (L.), European perch *Perca fluviatilis* L., northern pike *Esox lucius* L., tench *Tinca tinca* (L.), Wels catfish *Silurus glanis* L. and brown trout *Salmo trutta fario* L. Until recently, perch represented the numerically dominant species of the fish community that could be rather easily caught in the reservoir (S. Gécy, personal communication).

### 2.2. Fish and Parasite Collection

During the period 2009–2010, 43 perch (*P. fluviatilis*) were collected nearby the Jaklovce village situated at the mouth of the Hnilec River (Figure 1), which is regarded as the most seriously heavy metal-polluted site of the reservoir [23].

**Figure 2.**
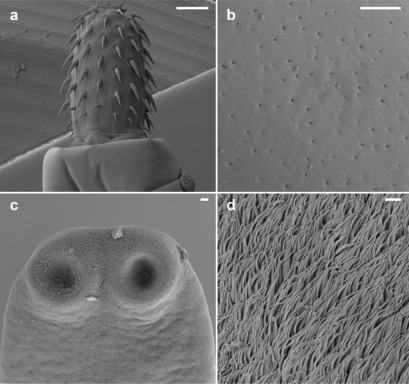
Scanning electron micrographs illustrating *Acanthocephalus lucii* (**a**,**b**) and *Proteocephalus percae* (**c**,**d**). a—proboscis with hooks; b—tegument with orifices of the lacunary system; c—scolex and d—tegument with microtriches. Scale-bars: a—100 μm; b, d—1 μm; c—10 μm.

Fish were transported in water from the reservoir to the laboratory, where they were killed by severing the spinal cord. The perch were sampled and dissected with the help of stainless steel instruments and MiliQ water. The fork length and wet weight of each fish was recorded prior to dissection. Samples of the muscles, kidney, liver, brain, hard roe (fully ripe internal egg masses in the ovaries) and soft roe (the testis filled with mature sperm) and the adipose tissue were taken from all individuals. These samples were stored individually in glass vials and deep frozen until posterior processing for trace element analysis. Fish digestive tracts were screened for parasites using a stereomicroscope. Acanthocephalans (*Acanthocephalus lucii*) and cestodes (*Proteocephalus percae*) (Figure 2(a–d)) representative of all infected fish were frozen separately for metal analysis. The adult intestine acanthocephalan *A. lucii* was detected in 35 perch indicating a prevalence of 82.3% and a mean intensity of 3 ± 7.9 (1–48) specimens. The cestode *P. percae* was detected in 28 perch indicating a prevalence of 64.7% with a mean intensity of 1.4 ± 1.63 (1–7) specimens in the 43 fish hosts studied. Two perch were free of infection. All specimens were isolated from each fish intestine, washed in ultrapure water, counted and frozen individually for further trace element analysis.

### 2.3. Experimental Design

The experiment was designed for a comparison of the metal uptake (cadmium) by two different parasite species. Live adult worms, two and four cestodes (*P. percae*) and acanthocephalans (*A. lucii*), respectively per exposure, obtained from perch intestine were several times washed in 0.9% neutral phosphate-buffered saline (PBS, pH 7–7.3) and then, in a laminar flow cabinet, immediately replaced into 50 mL sterile culture flasks with graduation (Sigma Aldrich, Germany) containing 20 mL RPMI-1640 medium (Sigma Aldrich, Germany) treated with antibiotics (100 U/mL penicillin, and 100 U/mL streptomycin) and kept at 16 ± 1 °C in a cool room for 1, 2 and 3 weeks. The cadmium solutions were prepared from a stock solution of 100 mg/L cadmium chloride (CdCl_2_). Twelve parasite sets (six acanthocephalans and six cestodes) were exposed to two different final concentrations of Cd^2+^: (i) 6.132 mg/L Cd^2+^ (high dose) and (ii) 0.6132 mg/L Cd^2+^ (low dose), while the remaining two sets served as unexposed acanthocephalan and cestode controls cultured only in RPMI-1640 for 3 weeks. The integrity and motility (spontaneous motoric activity without physical stimulation) of worms were monitored under stereomicroscope daily. At the end of each week, two groups of both parasite species (high dose; low dose) were rinsed with ultrapure water and frozen at −20 °C for subsequent cadmium analysis. The parasites from uncontaminated medium were processed in the same way at the end of the third week. During the experiment all parasites survived, and the integrity of their tissues was preserved.

### 2.4. Metal Analysis

Fish and parasite samples from the reservoir were analyzed for the presence of nine heavy metals (As, Cd, Cr, Cu, Hg, Mn, Ni, Pb and Zn). All materials used in the digestion process were thoroughly acid-rinsed. Samples were weighted (around 100–150 mg wet weight) and digested in Teflon vessels with 2 mL HNO_3_ (Merck, Suprapur) and 1 mL H_2_O_2_ (Panreac), at 90 °C in an oven and left overnight, according to the standard protocol used at the “Centres Científics i Tecnològics de la Universitat de Barcelona (CCiTUB)”. After the complete digestion, samples were diluted with 30 mL of Milli-Q water and then analysed for trace elements in an inductively coupled plasma mass spectrometer (Perkin Elmer Elan 6000). For quality assurance standard reference materials Dogfish (*Squalus acanthias*) liver (DOLT-3) and muscle (DORM-2) (National Research Council, Canada) were used in the analyses. Several analytical blanks were prepared under the same conditions and analyzed along with samples in order to determine the detection limits. Values of all monitored heavy metals are presented in μg·g^−1^ wet-weight.

### 2.5. Data Analysis

The data obtained did not meet the requirements for parametric statistical tests. Thus, they were analyzed non-parametrically. Adipose tissue and soft roe were excluded from the statistical analysis due to the insufficient number of samples.

The differences in heavy-metal concentrations among the analyzed tissues, between parasite and host tissues, and the two parasite species for each tissue separately were tested. For comparing two independent groups, Mann-Whitney U test was used, for multiple independent groups Kruskal-Wallis ANOVA with multiple comparisons of mean ranks for all groups [24] was selected. The analyses were performed in Statistica for Windows, version 9.0 [25].

The bioconcentration factors (BF) were calculated according to Sures *et al.* [16], as the ratio of the element concentration in the parasites to that in different host tissues (BF = C_[parasite]_/C_[host tissue]_) and checked for statistical significance with non-parametric Mann Whitney U test or Kruskal-Wallis ANOVA with multiple comparisons of mean ranks for all groups, so-called post hoc multiple comparing [24].

## 3. Results

### 3.1. Element Distribution in Host Tissues and Parasites

The detection limits (mean blank value plus three standard deviations of the mean blank) for each element and accuracy values are presented in Table 2.

Element concentrations detected in the seven tissue types of perch and the above mentioned two parasite species are presented in Table 3. Mean concentrations of heavy metals in fish decreased in the order zinc > copper > manganese > mercury > arsenic > chromium > cadmium > nickel > lead. Zinc was the dominant element in all of the evaluated fish tissues. Although the obtained concentrations of copper were also very high, they were always considerably lower than those of zinc found in the same organ, a fact that could confirm an antagonistic zinc-copper interaction. Considering the mean concentrations of accumulated heavy metals, the kidney was found to be a key target organ followed by the liver, adipose tissue, hard roe, brain, muscle and soft roe.

Results of the Kruskal-Wallis test indicated significant differences among heavy metal concentrations in individual tissue samples (P < 0.001). Most metals showed a strong tendency towards a specific tissue. This was evident in Ni and Zn with kidney concentrations respectively 7 to 35 times higher than those in the remaining tissues. The liver was the main site for Cu accumulation, while the highest quantity of As, Cr, Mn and Pb were found in adipose tissue. The level of Hg was up to 12-fold higher in the muscle tissue in comparison to those in other organs. Cadmium predominated in the liver and kidney.

Concentrations of individual heavy metals in perch tissues were mostly significantly lower than those in parasites. Only Hg was primarily concentrated in fish muscle (P < 0.05). Zinc was also found in higher quantity in the kidney, than in parasite tissues, but with a difference not statistically significant.

**Figure 3.**
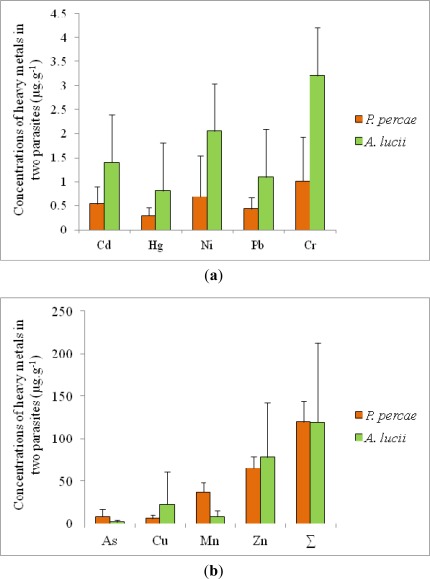
(**a**,**b**) Average concentrations (μg·g^−1^) of heavy metals in *Acanthocephalus lucii* (n = 35) and *Proteocephalus percae* (n = 28) parasitizing perch (*Perca fluviatilis*) from the Ružín water reservoir.

Comparing the two parasite species, the mean concentrations of the analyzed elements was found to be almost the same in the acanthocephalans and cestodes with values of 118.86 and 118.88 μg·g^−1^, respectively, but significant differences were found considering concentrations of individual elements (Figure 3(a,b)). Cestodes concentrated higher amounts of As and Mn (P < 0.001), while acanthocephalans accumulated preferentially Cd, Cu, Cr and Ni (P < 0.05) and Hg (P < 0.01). Based on this, the accumulation potential of the acanthocephalans was judged to be higher when compared to the cestode species.

**Figure 4.**
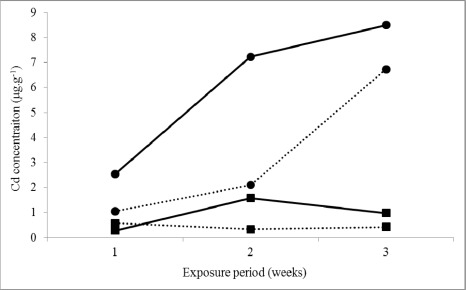
Uptake of cadmium by cestodes *Proteocephalus percae* (n = 6) and acanthocephalans *Acanthocephalus lucii* (n = 6). Legend: ▂▂▂ 6.132 mg/L Cd^2+^ (high dose); **••••••** 0.613 mg/L Cd^2+^ (low dose); • acanthocephalans, ▪ cestodes.

To prove correctness of our later assumption, we designed an experiment in which both parasite species were exposed to cadmium (better accumulated by acanthocephalans in field conditions), during 1, 2 and 3 weeks. The unexposed acanthocephalans and cestodes (controls) were kept in medium without any additive. Cd concentrations assessed in acanthocephalans and tapeworms were 0.08 μg·g^−1^ 0.049 μg·g^−1^, respectively. As shown in Figure 4, acanthocephalans accumulated Cd more intensively than cestodes, which is in accordance with present data obtained in field conditions.

### 3.2. Bioconcentration Factors (BFs)

The differences among most concentrations of heavy metals accumulated in the muscle, liver, kidney, brain and hard roe of perch and those in both parasites were statistically significant (Table 4). The highest BFs were obtained for Cd, which presented accumulation ratios between both parasites and fish organs (muscle, brain, hard roe) orders of magnitude higher than those obtained for other elements. The maximum BF of cadmium was stated for *A. lucii* and muscle of perch (BF = 267.6). There was a significantly higher (P < 0.001) accumulation capacity of Pb in both parasites in relation to fish organs. In *A. lucii*, mean Pb concentrations were approximately 52, 56, 67 and 170-times higher than those detected in muscle, brain, liver and hard roe of perch, respectively. Concentrations of Pb in *P. percae* attained similarly much higher levels comparing to fish organs (see Table 4).

## 4. Discussion

In aquatic ecosystems most pollutants tend to build up in organisms, in which they can reach levels hundreds or thousands times higher than the respective water levels [26]. Fish that are close to the top of the aquatic food web and have a relatively long life span, concentrate high amounts of pollutants (among them heavy metals) and, therefore, they are widely used as biological indicators [27]. Some authors (e.g., [28]) showed in their studies that the target tissues of heavy metals in fish are those metabolically more active, accumulating high levels of them, such as the liver, kidneys and gills. The liver serves as a storage site for heavy metals, and it plays an important role in detoxication and elimination of many harmful substances from the body [29,30]. In the present study, Cu reached 5–21 times higher concentrations in the liver compared to the other fish tissues. However, considering the overall quantity of heavy metals the liver of perch was proved only as the second most important heavy metal-storage organ.

It has been found that the kidney was the key target organ receiving the largest quantities of heavy metals in perch. It is characterized as a metabolically active and eliminative organ that performs many vital functions including the removal of heavy metals from the body [31]. High levels of some trace elements (Cd, Ni, and Zn) found in this organ in the present study might also suggest this function. In fact, present findings are in agreement with data obtained by Pourang [32] and Giguére *et al.* [33] who also found the kidney of pike (*Esox lucius*) and yellow perch (*Perca flavescens*) as the most important storage organ for Zn.

A number of ecotoxicological reports confirmed that fish muscle represents one of the least metal-containing tissues [34,35]. Nevertheless, fish meat serves as an excellent source of proteins for humans and due to that the monitoring of fish muscle is of great importance. In the present study significantly higher concentrations of mercury have been found in muscle of perch from the Ružín water reservoir compared to the remaining analyzed fish organs. Concentrations of Hg in muscle (1.05 μg·g^−1^ wet wt) exceeded more than two-times the maximum mercury level admitted in foodstuffs in European countries [36]. Similar results were obtained by Farkas *et al.* [35] who studied heavy metal concentrations in freshwater bream (*Abramis brama*, L.) in Lake Balaton (Hungary). The gills and liver accumulated the highest metal concentrations, except mercury, which was detected in highest levels in fish muscle. One of the possible reasons of the highest mercury level in muscle is the high content of functional proteins in muscle, having high affinity to this element [37]. Since Hg is known as a human toxic agent, which primary source is through eating fish [38], the present data are significant in terms of potential health impacts on inhabitants of the region. Long-term consumption of contaminated fish meat from the Ružín water reservoir mainly by fishermen and their families could cause hydrargyrism, which can cause alterations of the nervous system, brain damage, induction of reproductive diseases and many other disorders.

Several earlier studies performed in freshwater ecosystems showed that intestinal parasites, mostly acanthocephalans, can accumulate heavy metals at concentrations that are orders of magnitude higher than those in their fish hosts and thus, they could provide valuable information about the chemical state of the environment [10,39,40]. In present study, it was confirmed that acanthocephalans as well as cestodes seem to be good indicators of environmental conditions. The reasonable argument for this could provide our results of cadmium accumulation by parasites. Although the concentrations of cadmium in water and bottom sediments of the reservoir were low (0.00016 mg·L^−1^and 1.4 mg·kg^−1^ dry weight) in comparison to other metals, those in *P. percae* and *A. lucii* were relatively high (0.54 and 1.39 μg·g^−1^). It implies that even very low concentration of metals in environment can be detected using parasites possessing extraordinary accumulation capacity. Because about 2.5-times higher concentrations of Cd were detected in *A. lucii* than *P. percae*, we believe that acanthocephalans might be better biomonitors of heavy metals. Our results from field study were confirmed under experimental conditions, in which *A. lucii* accumulated Cd approximately 9-times more than *P. percae* (8.50 and 0.98 μg·g^−1^ wet·wt, respectively; three-week, high Cd dose).

The bioconcentration factors obtained for almost all metals confirmed the high accumulation capacity of parasites (*A. lucii* and *P. percae*) compared also to fish tissues. The BFs (C_[parasite]_/C_[muscle]_) are of particularly great importance denoting much higher detection ability of parasites than fish muscle, which is the tissue tested more commonly in heavy metal biomonitoring studies. In present study the highest bioconcentration factors were determined for cadmium in A*. lucii* parasites and muscle and hard roe of perch. Similar results achieved Jankovská *et al.* [41], who studied concentrations of Zn, Mn, Cu and Cd in different tissues of the same fish species and *A. lucii* in the Jevanský stream near Prague (Czech Republic). A potential of cadmium to accumulate in muscles and gonads determined by bioconcentration factors (194 and 110, respectively) was also very high. Slightly different results reported Sures *et al.* [16] in his study on accumulation of 17 elements in perch tissues and *A. lucii* parasites. The highest bioconcentation factors were assessed for copper in muscle (250) and liver (25) of fish. Our data, for a comparison, were lower; BFs for Cu were only 67.7 (muscle) and 3.6 (liver).

Among the nine elements analysed in our study, *A. lucii* accumulated Cd, Cu, Hg, Cr and Pb in significantly higher concentrations compared to cestodes. On the other hand, *P. percae* accumulated more intensively the elements As and Mn. Species-specific preference for some metals was also shown in the paper by Turčeková *et al.* [40]. Similarly to our data, they found that cestodes accumulated higher concentrations of As and lower quantities Cd than acanthocephalans, but a reliable explanation of this phenomenon could be a question for further laboratory experiments and more detailed ultrastructural observations are required.

The present study was built upon our earlier work performed at the same Ružín water reservoir by Turčeková *et al.* [40]. Recent data have demonstrated a decrease in concentration of almost all monitored elements. In fish muscles, the main decrease of contamination was observed for Cu (1.7 μg·g^−1^ and 0.32 μg·g^−1^) and Pb (0.07 μg·g^−1^ and 0.02 μg·g^−1^), which are the two major pollutants coming from the former mining activities. Lead concentration in the fish liver also decreased (0.08 μg·g^−1^ and 0.02 μg·g^−1^) but Cu remained at similar level in both periods. The amount of heavy metals in parasites was also significantly lower in the present study. In acanthocephalans, the highest decrease was detected again for Cu and Pb. Nevertheless, increased mercury concentrations in the fish muscle were found (see above) and, therefore, continuing monitoring is recommended. In addition, high concentrations of arsenic were detected in the muscles and liver of perch, and in the parasites. Since arsenic was produced as a by-product of refining the ores of other metals, such as copper and lead in the erstwhile factories, so it could be assumed that present high concentration of this element estimated in fish tissues and parasites still come from these old industry activities but it is difficult to provide a satisfactory explanation for this arsenic increase.

On the whole, comparison of results from both sampling periods seems to point out that heavy metal burden of the Ružín water reservoir appears to be decreasing and a gradual recovery of this ecosystem is taking place in the course of time.

## Figures and Tables

**Table 1.** Content of heavy metals in water (mg·L^−1^) and sediments (mg·kg^−1^ dry weight) of the Ružín water reservoir.

	**As**	**Cd**	**Cr**	**Cu**	**Hg**	**Ni**	**Pb**	**Zn**
Water ^1^	0.0041	0.00016	0.0013	0.0097	0.00005	0.0038	0.0055	0.02
Sediments ^2^	57.9	1.4	91.6	382.0	1.9	63.2	78.8	517.0

Data provided by

1 Slovak Water Management Company, Košice;

2 Hiller *et al.* [21].

**Table 2.** Detection limits (ng·mL^−1^) in the standard reference material DORM-2 and DOLT-3 determined by inductively coupled mass spectrometry (ICP-MS).

	**Detection limit**	**Standard**	**ICP-MS value Mean (±SD)**	**Certified value Mean (±CI)**	**Accuracy (%)**
As	<0.01	DORM-2	18.65 (0.21)	18.00 (1.10)	103.6
Cd	<0.01	DOLT-3	18.10 (0.44)	19.40 (0.60)	93.3
Cr	0.63	DORM-2	34.40 (0.21)	34.70 (5.50)	98.3
Cu	0.16	DOLT-3	28.88 (0.30)	31.20 (1.0)	92.6
Hg	0.07	DORM-2	4.96 (0.07)	4.64 (0.26)	106.9
Mn	0.15	DORM-2	3.35 (0.28)	3.66 (0.34)	91.5
Ni	0.13	DOLT-3	2.60 (0.11)	2.72 (0.35)	95.6
Pb	0.03	DORM-2	0.06 (0.01)	0.07 (0.01)	96.9
Zn	3.73	DORM-2	23.91 (1.18)	25.60 (2.30)	93.4

±SD, standard deviation; ±CI, confidence interval.

**Table 3.** Trace element concentrations in perch tissues and parasites *A. lucii* an *P. percae* (μg·g^−1^) and SD values from the Ružín water reservoir.

		**Muscle**	**Liver**	**Kidney**	**Adipose tissue**	**Soft roe**	**Hard roe**	**Brain**	***A. lucii***	***P. percae***

		(n = 43)	(n = 43)	(n = 43)	(n = 8)	(n = 14)	(n = 27)	(n = 43)	(n = 35)	(n = 28)
As	Mean	0.19	0.802	0.965	1.37	0.385	0.218	0.419	2.05	7.99
	(±) SD	0.092	0.365	0.352	0.907	0.093	0.100	0.181	2.49	8.94
Cd	Mean	0.0050	1.31	1.33	0.139	0.022	0.0073	0.011	1.39	0.540
	(±) SD	0.0044	2.06	1.58	0.142	0.034	0.0027	0.013	1.67	0.364
Cr	Mean	0.439	0.423	0.585	1.65	0.381	0.459	0.536	3.21	5.89
	(±) SD	0.148	0.087	0.147	0.629	0.071	0.105	0.109	6.25	0.911
Cu	Mean	0.319	6.81	2.29	0.944	0.304	0.595	1.46	22.2	1.01
	(±) SD	0.120	4.31	0.548	0.905	0.063	0.181	0.268	38.25	3.93
Hg	Mean	1.05	0.739	0.801	0.187	0.305	0.087	0.404	0.810	0.290
	(±) SD	0.379	0.459	0.396	0.161	0.187	0.040	0.227	0.713	0.166
Mn	Mean	0.252	2.83	2.36	8.17	0.207	1.60	0.400	8.22	37.2
	(±) SD	0.087	0.893	1.81	9.66	0.276	0.573	0.197	6.39	10.6
Ni	Mean	0.147	0.286	1.103	0.628	0.231	0.199	0.556	2.05	0.680
	(±) SD	0.119	0.335	3.14	1.25	0.110	0.152	0.839	3.87	0.867
Pb	Mean	0.019	0.016	0.098	0.114	0.0082	0.0072	0.017	1.09	0.430
	(±) SD	0.039	0.014	0.086	0.202	0.006	0.005	0.030	2.72	0.235
Zn	Mean	7.50	25.8	281.0	20.0	7.74	18.6	13.6	78.1	64.9
	(±) SD	1.97	3.22	99.4	18.8	4.01	1.34	3.77	64.7	14.0

**Table 4.** Mean bioaccumulation factors (BFs) calculated for the analyzed elements in *A. lucii* and *P. percae* and respective perch tissues.

		**As**	**Cd**	**Cr**	**Cu**	**Hg**	**Mn**	**Ni**	**Pb**	**Zn**
*A. lucii*	Muscle	9.9 ***	267.6 ***	7.4 ***	67.7 ***	0.8	31.2 ***	11.8 ***	52.1 ***	10.1 ***
*P. percae*		37.2 ***	128.2 ***	2.2 **	19.8 ***	0.3 ***	149.0 ***	4.1*	21.3 ***	8.7 ***
*A. lucii*	Liver	2.8	1.0	7.8 ***	3.6	1.1	2.9	6.6 ***	67.4 ***	3.0
*P. percae*		9.2 **	0.6	2.4 **	0.9	0.5 **	12.2 *	2.1	28.6 ***	2.5
*A. lucii*	Kidney	2.1	0.9	5.8 *	9.2 **	0.9	4.1 **	1.3	12.3	0.3
*P. percae*		8.1 *	0.5	1.8	2.5	0.4 ***	18.1 ***	1.0	6.1	0.2
*A. lucii*	Brain	4.2 ***	107.5 ***	6.0 *	14.5 ***	1.9	20.9 ***	3.6	55.7 ***	5.9 ***
*P. percae*		15.9 ***	56.2 *	1.8	3.9 ***	0.8	101.2 ***	0.8	19.1 ***	4.6 ***
*A. lucii*	Hard roe	8.7 ***	191.0 ***	7.6 ***	36.8 ***	8.7 ***	5.1 ***	8.1 ***	170.7 ***	4.3 **
*P. percae*		32.1 ***	72.1 **	2.3 *	10.7 ***	3.2	24.1 ***	2.6	70.3 ***	3.6 **

* p < 0.05;

** p < 0.01;

*** p < 0.001.
